# Biochemical Methanol Gas Sensor (MeOH Bio-Sniffer) for Non-Invasive Assessment of Intestinal Flora from Breath Methanol

**DOI:** 10.3390/s21144897

**Published:** 2021-07-19

**Authors:** Koji Toma, Kanako Iwasaki, Geng Zhang, Kenta Iitani, Takahiro Arakawa, Yasuhiko Iwasaki, Kohji Mitsubayashi

**Affiliations:** 1 Department of Biomedical Devices and Instrumentation, Institute of Biomaterials and Bioengineering, Tokyo Medical and Dental University (TMDU), 2-3-10 Kanda-Surugadai, Chiyoda-ku, Tokyo 101-0062, Japan; toma.bdi@tmd.ac.jp (K.T.); i.bdi@tmd.ac.jp (K.I.); arakawa.bdi@tmd.ac.jp (T.A.); 2 Graduate School of Medical and Dental Sciences, Tokyo Medical and Dental University (TMDU), 1-5-45 Yushima, Bunkyo-ku, Tokyo 113-8510, Japan; ma170016@outlook.jp (K.I.); ma201102@tmd.ac.jp (G.Z.); 3 Faculty of Chemistry, Materials and Bioengineering, Kansai University, Osaka 564-8680, Japan; yasu.bmt@kansai-u.ac.jp

**Keywords:** intestinal flora, methanol, gas-phase biosensor, enzymatic cascade reaction, fluorescence

## Abstract

Methanol (MeOH) in exhaled breath has potential for non-invasive assessment of intestinal flora. In this study, we have developed a biochemical gas sensor (bio-sniffer) for MeOH in the gas phase using fluorometry and a cascade reaction with two enzymes, alcohol oxidase (AOD) and formaldehyde dehydrogenase (FALDH). In the cascade reaction, oxidation of MeOH was initially catalyzed by AOD to produce formaldehyde, and then this formaldehyde was successively oxidized via FALDH catalysis together with reduction of oxidized form of β-nicotinamide adenine dinucleotide (NAD^+^). As a result of the cascade reaction, reduced form of NAD (NADH) was produced, and MeOH vapor was measured by detecting autofluorescence of NADH. In the development of the MeOH bio-sniffer, three conditions were optimized: selecting a suitable FALDH for better discrimination of MeOH from ethanol in the cascade reaction; buffer pH that maximizes the cascade reaction; and materials and methods to prevent leaking of NAD^+^ solution from an AOD-FALDH membrane. The dynamic range of the constructed MeOH bio-sniffer was 0.32–20 ppm, which encompassed the MeOH concentration in exhaled breath of healthy people. The measurement of exhaled breath of a healthy subject showed a similar sensorgram to the standard MeOH vapor. These results suggest that the MeOH bio-sniffer exploiting the cascade reaction will become a powerful tool for the non-invasive intestinal flora testing.

## 1. Introduction

Intestinal flora has been gaining its attention year by year after several reports were made regarding influences of the intestinal flora to general health conditions [1,2]. In particular, two dominant bacteria, the *Bacteroidetes* and the *Firmicutes* [3,4,5,6], play important roles as their composition in intestinal bacteria, and metabolites affect the host’s immune function, obesity, drug metabolism, and absorption [7,8,9].

Currently, there are two main methodologies to assess the status of intestinal flora: culture method and genome analysis. In culture method, bacteria extracted from stool sample are cultured, and the resultant morphology and color tone of the colonies are observed [10,11]. This method is inexpensive, but one needs a long time to have results. Furthermore, it is unable to observe more than 90% of intestinal microorganisms because of lack of growth substrates and difficulties in maintaining the culture environment such as temperature, pH, and osmotic pressure, which are varied for different bacteria [12]. For example, it is hard for obligatory anaerobic bacteria such as *Bacteroidetes* to survive and grow in healthy conditions if the stool sample is exposed in ambient air for a long time. Therefore, this method is suitable for detecting the presence of bacteria of interest in a stool sample that one can grow, but not for reflecting actual intestinal flora nor for quantitative assessment.

On the other hand, in genome analysis with next generation sequencing, it is possible to obtain nucleotide sequence information rapidly along with high-throughput without the culturing. This method identifies sequential information of 16S ribosomal RNA (rRNA), which is amplified using polymerase chain reaction (PCR). The obtained sequential information allows phylogenetic classification and identification of bacteria. A report said 93% of 1822 16S rRNA sequences from intestine bacteria that are detectable by the genome analysis cannot be identified by the cultured method [13]. The disadvantage of this method is the cost through molecular phylogenetic processes [14].

Methanol (MeOH) is produced from the degradation of dietary fiber, pectin, and by intestinal bacteria, including *Bacteroidetes* [15,16,17]. This produced MeOH is transferred to the blood stream, and a fragment of the blood MeOH is released through exhaled breath. The study of Laakso et al. showed a good correlation of the MeOH concentration between exhaled breath and blood [18]. There are some reports demonstrating increase in MeOH concentration in exhaled breath after intaking pectin. For example, a study by Dorokhov et al. observed the increase of the blood MeOH concentration up to 1.5 times after eating vegetables [19]. According to a study by Lindinger et al., eating pectin-contained fruits, such as apples, lead to the increase in breath MeOH concentration [20]. These reports suggest the potential for non-invasive assessment of intestinal bacteria activity by measuring MeOH in exhaled breath.

MeOH in exhaled breath has been analyzed by various methodologies, such as gas-liquid chromatography [21], proton-transfer-reaction mass spectrometry (PTR-MS) [20], and selected-ion flow-tube mass spectrometry (SIFT-MS) [22,23]. Despite their capability of identifying MeOH in gas mixture, the size and complexity of the system are drawbacks for the on-site MeOH assessment.

On the other hand, sensors are expected to be used closer to the site than lab-based analytical devices. Therefore, it is required that sensors be simple to operate and compact in size along with sufficient sensitivity, selectivity, and capability of continuous measurement for some occasions [24,25,26,27,28,29]. However, generally, there is a trade-off between being compact in size and having high sensitivity, and it is very challenging to meet both requirements. To overcome these challenges, a lot of efforts have been devoted to improving the sensitivity and selectivity of MeOH gas sensors. For example, Li et al. synthesized a Pt-SnO_2_ nanoparticles composite for a chemoresistive gas sensor [30]. An alumina tube surface was coated with this nanocomposite to fabricate a sensor, and MeOH vapor was measured by changes in the resistance of the sensor. As a result, the sensor using Pt-SnO_2_ showed about 10-fold better sensor output to 100 ppm methanol vapor at 120 °C lower operating temperature than those with pristine SnO_2_ microspheres (220 °C). As an another SnO_2_-based chemoresistive gas sensor, Chen et al. developed a lanthanum-doped SnO_2_ nanocomposite. Similar to Li’s study, the lanthanum-doped SnO_2_ nanocomposite was coated on a ceramic tube, and MeOH vapor was measured by changes in the resistance. The sensor output was improved by a factor of 7 with the lanthanum-doped SnO_2_ compared to the sensor with only SnO_2_ when measuring 75 ppm MeOH vapor at the operating temperature of 220 °C [31]. A MeOH gas sensor employing metal-organic framework was reported by Andrés et al. [32]. The sensor was fabricated by depositing MIL-96(Al) nanoparticles on a Si/SiO_2_ substrate with interdigitated electrodes. It showed different sensor outputs to water and MeOH vapor from other VOCs, which will be useful to identify these two components out of gas mixture. There are also reports regarding handheld MeOH gas sensors for immediate use in the field [18,33,34]. Broek et al. introduced a MeOH sensor that was composed of a separation column and Pd-doped SnO_2_ chemoresistive sensor. This combination made the size compact and made it possible to measure MeOH vapor even in the presence of much higher concentration of ethanol within 2 min [33]. As another approach, recent advances in machine learning were applied to an electronic nose (e-nose) with a pattern recognition algorithm. Due to their high technological affinity, the e-nose using graphene FET was able to classify water, MeOH, and ethanol with high accuracy [35].

Although there has been great advancement of such sensors, it is not yet easy to selectively measure MeOH. Recently, we have developed a liquid phase biosensor for MeOH (the AOD-FALDH fluorosensor) that employed two-enzyme cascade reaction with alcohol oxidase (AOD), formaldehyde dehydrogenase (FALDH), and fluorometry [36]. The cascade reaction begins with oxidation of MeOH via AOD to produce formaldehyde, and then produced formaldehyde is oxidized via FALDH together with reduction of a coenzyme, which is an oxidized form of β-nicotinamide adenine dinucleotide (NAD^+^). MeOH was measured by detecting autofluorescence from a reduced form of NAD (NADH). Due to the cascade reaction, the AOD-FALDH fluorosensor was able to measure MeOH with high selectivity, especially from other aliphatic alcohols, including ethanol. Based on these results, it is expected that the AOD-FALDH fluorescence is applicable to the non-invasive measurement of MeOH in such body fluids as urine or saliva, but it would be quicker and easier if the same assessment could be made with exhaled breath. As mentioned above, the measurement of gas phase samples is more challenging than liquid phase samples because gas phase samples are usually invisible and more rapidly diffuse. In particular, exhaled breath contains a considerable portion of water with the relative humidity of 65–91%, according to the study by Mansour et al. [37]. Besides, the MeOH concentration in breath is approximately 3000-times lower than that in blood [18]. Therefore, a sensor for MeOH in exhaled breath needs to be highly sensitive to MeOH as well as insensitive to humidity. Our biochemical gas sensor (bio-sniffer) has shown those required characteristics in previous studies for ethanol, acetaldehyde, isopropanol, and acetone [38,39,40,41], and in this study, we combined the advantages of the bio-sniffer and AOD-FALDH cascade reaction to measure MeOH in exhaled breath.

## 2. Materials and Methods

### 2.1. Materials and Reagents

Alcohol oxidase (AOD, A2404-1KU, from *Pichia pastoris*) was obtained from Sigma-Aldrich Japan (Tokyo, Japan). Formaldehyde dehydrogenase (FALDH, from *Pseudomonas* sp.) was from two different manufacturers, Funakoshi (Tokyo, Japan) and Toyobo (Osaka, Japan). An oxidized form of β-nicotinamide adenine dinucleotide (NAD^+^) was purchased from Oriental Yeast (Tokyo, Japan). A hydrophilic polytetrafluoroethylene (H-PTFE) membrane (thickness: 80 μm, porosity: 80%, pore size: 0.2 μm) was from Millipore (Burlington, MA, USA). Poly [2-methacryloyloxyethyl phosphorylcholine (MPC)-co-2-ethylhexyl methacrylate (EHMA)] (PMEH) was synthesized in house by the free radical-polymerization method [42]. Polyethyleneimine (PEI, average molecular weight of 10,000, 164-17821) and glutaraldehyde (GA, 079-00533) were purchased from Fujifilm Wako Pure Chemical (Osaka, Japan).

All reagents for the following buffer solutions were obtained from Fujifilm Wako Pure Chemical (Osaka, Japan). For the phosphate buffer (PB, 100 mM) solution, we added potassium dihydrogen phosphate solution (100 mM in ultrapure water) to disodium hydrogen phosphate solution (100 mM in ultrapure water) to buffer the solution pH from 6.5 to 8.5. For the Tris-HCl solution (100 mM), we added HCl solution to 100 mM trimethylolaminomethane solution to buffer the pH from 8.5 to 10.5.

### 2.2. Construction of MeOH Bio-Sniffer

**Figure 1:**
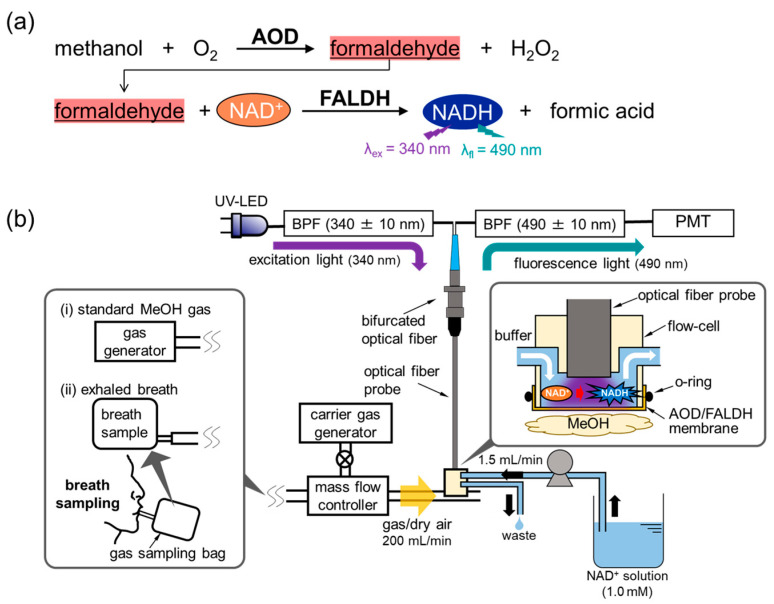
(**a**) Measurement principle, and (**b**) a schematic illustration of the MeOH bio-sniffer.

The MeOH bio-sniffer exploited a cascade reaction with AOD and FALDH that is described elsewhere (Figure 1a) [36]. The reaction begins with oxidation of MeOH via AOD catalysis, which produces formaldehyde. Then, oxidation of the produced formaldehyde occurs via FALDH in conjunction with the reduction of NAD^+^, in which formic acid and NADH are produced. We measured MeOH vapor indirectly by autofluorescence of NADH, whose emission *λ*_em_ and excitation *λ*_ex_ wavelengths were 490 nm and 340 nm, respectively. In this principle, the fluorescence intensity increased if the concentration of MeOH increased.

The device configuration of the MeOH bio-sniffer was similar to the ones in our previous studies except for the membrane [39,43,44]. Briefly, it consisted of a bifurcated optical fiber (PVSMA2-2 STU600-STUH190S, Mitsubishi Cable Industries, Tokyo, Japan), an optical fiber probe (F1000-900, Ocean Insight, Orlando, FL, USA), a UV light emitting diode (UV-LED, UF4LU-0GD01, DOWA, Tokyo, Japan), and a photomultiplier tube (PMT, C9692-11, Hamamatsu Photonics, Shizuoka, Japan). At the end of the optical fiber probe that was connected to the bifurcated optical fiber, a flow-cell made from poly methyl methacrylate was attached. An AOD-FALDH membrane was attached on the flow-cell and worked as a gas-liquid diaphragm (Figure 1b). In the flow-cell, NAD^+^ solution flowed over the AOD-FALDH membrane at the flow rate of 1.5 mL/min using a peristatic pump (MP-1000-H, Tokyo Rikakikai, Tokyo, Japan). When MeOH vapor reached and diffused into the membrane, the enzymatic reaction described in Figure 1a occurred, which resulted in producing NADH in the solution. The excitation UV light, which originated from the LED and traveled through the bifurcated optical fiber and the optical fiber probe, was irradiated to NADH in the solution, and then emitted fluorescence from NADH was collected by the optical fiber. After being filtered by a bandpass filter (BPF, λ = 490 ± 5 nm, Asahi Spectra, Tokyo, Japan), the fluorescence light was detected by the PMT.

### 2.3. Preparation of AOD-FALDH Membrane

**Figure 2:**
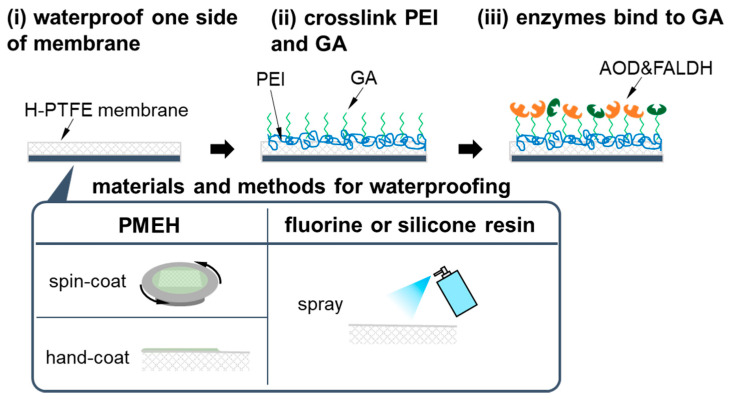
Preparation processes of the AOD-FALDH membrane.

First, waterproofing was made on the gas phase side of the membrane, which was opposite to the side of AOD and FALDH being immobilized, because the AOD-FALDH membrane was hydrophilic, and thus, the NAD^+^ solution in the flow-cell had leaked into the gas phase (Figure 2). In order to avoid both sides of a H-PTFE membrane becoming hydrophobic, the H-PTFE membrane was moderately wetted with ultrapure water before the treatment. Then, a surface of the H-PTFE membrane was coated with a hydrophobic material. After the coating, the H-PTFE membrane was dried out, and the membrane being hydrophobic on onside and hydrophilic on the other side was obtained. In the waterproof treatment, three different materials, including PMEH, a fluorine resin (Amedas, Columbus, Tokyo, Japan), and a silicone resin (NeverWet, Lancaster, PA, USA), were compared.

PMEH was either spin-coated or hand-coated. In the case of spin-coating, a 2 × 2 cm^2^ H-PTFE membrane wetted with ultrapure water was fixed on a silicon wafer, and then it was set on a spin-coater (1H-D7, Tokyo, Mikasa, Japan). After adding PMEH solution (10 *w*/*w*% in ethanol) dropwise to the H-PTFE membrane, it was spin-coated at spin rate of 4000 rpm. In the hand-coating, the PMEH solution applied to the H-PTFE membrane was spread out well by a hand. The coating with the fluorine resin or silicone resin was carried out by spraying a resin to a H-PTFE membrane for 5 s and drying it for 30 min.

Next, AOD and FALDH were immobilized on this waterproofed H-PTFE membrane with the similar protocol that has been described in detail elsewhere [36]. AOD and FALDH were crosslinked to PEI using GA on the hydrophilic side of a H-PTFE membrane in the following procedures. We first dropped 300 µL PEI solution (1 *w*/*w*% in PB) onto a 2 × 2 cm^2^ H-PTFE membrane and gently shook it for 5 min. Then, we rinsed excessive PEI from the membrane with 300 µL PB for three times. Afterwards, we dropped 300 µL GA solution (2.5 *v*/*v*% in PB) to the membrane and gently shook it for 5 min to crosslink PEI and GA. After rinsing excessive GA from the membrane with PB for three times, a mixed solution of AOD (2.0 unit/cm^2^) and FALDH (1.0 unit/cm^2^) was casted over the membrane and left for 15 min in a dark cold place (4 °C) to immobilize them. Finally, Tris-HCl solution was used to passivate the residual aldehyde group of GA, which would cause non-specific binding of NADH to GA.

### 2.4. Measurement of Standard MeOH Vapor and Exhaled Breath MeOH

First, the developed MeOH bio-sniffer was characterized with standard MeOH vapor. The standard MeOH released from a gas generator (PD-1B-2, Gastec, Kanagawa, Japan) was pumped to the flow-cell of the bio-sniffer at the regulated flow rate of 200 mL/min by a mass flow controller (FD-C1, Keyence, Osaka, Japan). The measurement began with acquiring the baseline of the fluorescence intensity from air cleaned through an active carbon filter (3001-17201, GL Sciences, Tokyo, Japan) and a silica gel filter (3001-17111, GL Sciences, Japan). This filtered air was also used as a carrier gas for MeOH vapor. Then, the gas line was switched to the gas generator, and MeOH vapor flowed to the bio-sniffer for 5 min. Finally, the gas line was switched back to the filtered air to initialize the measurement.

After the series of characterizations, the bio-sniffer was applied to the measurement of MeOH in exhaled breath of a healthy subject. This experiment was approved by the ethics committee of Institute of Biomaterials and Bioengineering, Tokyo Medical and Dental University (approval number: B2014-001), and was performed in accordance with the guidelines and regulations after informed consent had been obtained from a subject. Exhaled breath was collected from a subject who had been fasting for 2 h before the experiment and had administered no alcohol and medicine for three days prior to the experiment. The exhaled breath was sampled through the end-expiratory sampling. A subject drew in a deep breath though the nose and stopped for 10 s. Then, the subject breathed out through a mouth gently for 3 s to discard the dead space, followed by exhaling into a gas sampling bag. In the breath measurement, we pumped the breath sample to the MeOH bio-sniffer at the flow rate of 200 mL/min using a diaphragm pump (DSA-2-12, Denso Sangyo, Tokyo, Japan).

## 3. Results and Discussion

### 3.1. Optimization of AOD-FALDH Membrane

First, FALDH from different manufacturers were compared by the AOD-FALDH fluorosensor. The details of the AOD-FALDH fluorescence was described in the previous study [36]. Briefly, the AOD-FALDH membrane was attached at the end of the optical fiber probe that was connected to a bifurcated optical fiber (BIF600-UV/VIS, Ocean Optics, USA). Two ends of the bifurcated fiber were connected to the same UV-LED and PMT with the MeOH bio-sniffer. The fiber probe with the AOD-FALDH membrane was immersed in NAD^+^ solution (20 mM in pH8.5 PB) to excite NADH that was produced from the cascade reaction of MeOH with AOD and FALDH. Please note that the AOD-FALDH membrane used in this liquid phase fluorosensor had no waterproof treatment.

**Figure 3:**
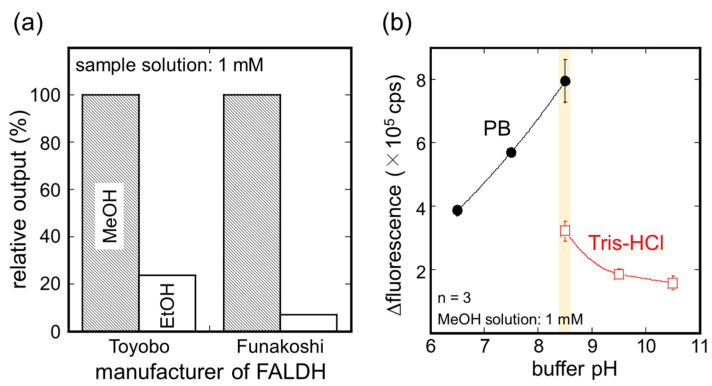
(**a**) Comparison of sensor output to MeOH and ethanol solutions using two different FALDH. (**b**) Influences of buffer solution pH on the activity of the AOD-FALDH membrane. Both if the experiments were carried out by the AOD-FALDH fluorosensor in the liquid phase.

Figure 3a shows a comparison of the sensor output of the AOD-FALDH fluorosensor to 1 mM MeOH or ethanol solution. For the AOD-FALDH membrane using FALDH from Funakoshi, the relative sensor output to ethanol solution was about 3 times lower than that from Toyobo, indicating that higher selectivity to MeOH can be obtained using FALDH from Funakoshi. This difference may be attributed to variations of bacteria and manufacturing conditions between two manufacturers. Although both FALDH were from *Pseudomonas* species, at present, there are more than 180 validly named species of *Pseudomonas* [45]. Therefore, it is supposed that activity and specificity of enzymes from different manufacturers are different. Based on this result, we decided to use FALDH from Funakoshi to prepare the membrane in subsequent experiments.

Next, the optimum pH for the AOD-FALDH membrane was investigated using the AOD-FALDH fluorosensor. The pH of the buffer solutions containing NAD^+^ was changed from 6.5 to 10.5 using PB and Tris-HCl (Figure 3b). MeOH solution was dropped into the buffer solution to make 1 mM MeOH solution while the probe of the AOD-FALDH fluorosensor was immersed in the buffer solution. As a result of three trials, the fluorescence output from 1 mM MeOH solution increased with increasing pH and became the maximum with pH 8.5 PB solution. Although the fluorescence output from Tris-HCl solution at pH 8.5 was lower than that with PB, it showed the maximum at pH 8.5 and decreased with increasing pH. These results indicated that using PB solution at pH 8.5 would allow for maximizing the catalytic activity of the AOD-FALDH membrane, and therefore, we decided to use pH 8.5 PB solution in subsequent experiments.

**Figure 4:**
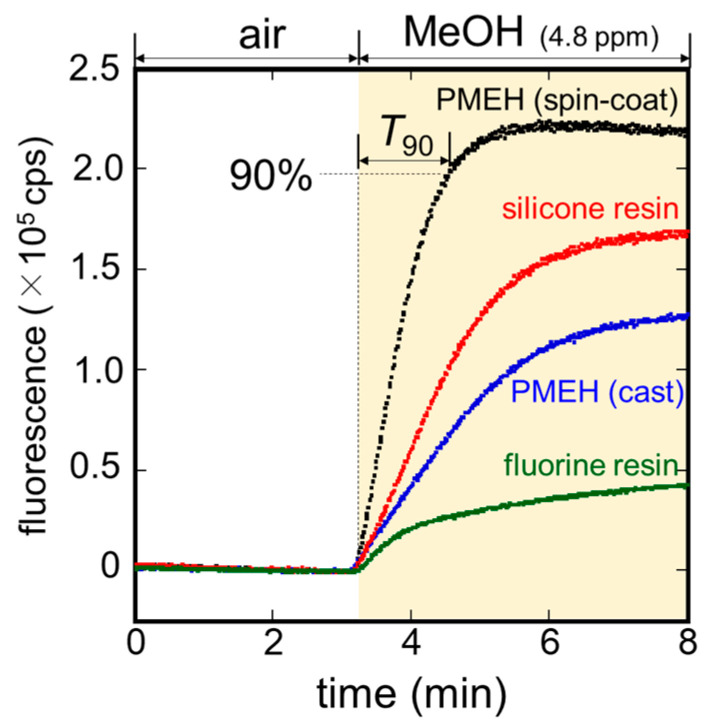
Sensor responses to 4.8 ppm MeOH vapor from the MeOH bio-sniffer, which employed four different waterproof treatments (spin-coating PMEH, casting PMEH, and spraying silicone or fluorine resins) on the gas-phase side of the AOD-FALDH membrane.

The influences of waterproof materials on the AOD-FALDH membrane were investigated. Except for the membrane coated with fluorine resin, no water leakage occurred. Figure 4 shows sensor responses to 4.8 ppm MeOH vapor by the MeOH bio-sniffer using differently waterproofed AOD-FALD membranes. All types of membranes resulted in the increase in the fluorescence from NADH upon applying MeOH vapor. Among them, the membrane waterproofed by spin-coating PMEH showed the largest sensor output along with a superior response represented by the time to reach 90% of the stable value (*T*_90_). The membrane prepared by spin-coated PMEH exhibited *T*_90_ of 91 sec, which was more than twice better than the others: casted PMEH, 229 sec; fluorine resin, 295 sec; silicone resin, 195 sec. Based on the all abovementioned aspects, we decided to waterproof the gas-phase side of the AOD-FALDH membrane by spin-coating PMEH.

### 3.2. Sensitivity to MeOH Vapor

**Figure 5:**
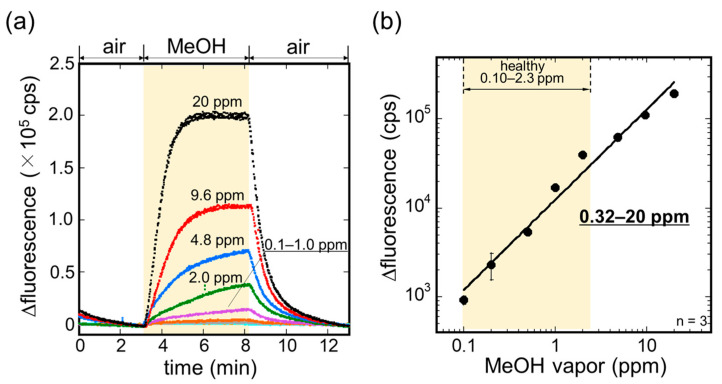
(**a**) Sensor outputs to various concentrations of MeOH vapor. (**b**) A calibration curve of the bio-sniffer to MeOH vapor.

The sensitivity of the MeOH bio-sniffer to MeOH vapor was investigated. The sensorgrams of the bio-sniffer to various concentrations of MeOH vapor are shown in Figure 5a. Following the stable baseline in air, the fluorescence intensity increased as a response to MeOH vapor. The increment in the fluorescence intensity was different depending on the concentration of MeOH vapor, suggesting the sensor output and the MeOH vapor concentration have a correlation. After demonstrating the measurement three times for each concentration, we summarized the sensor output, which was obtained by averaging fluorescence intensity for the last 30 s of the MeOH vapor application, as a function of the concentration of MeOH vapor in Figure 5b. A fitting curve of the plot was made by the following equation:

Δ*FI* (cps) = *A* [MeOH vapor (ppm)])*^B^* (1)

where *A* = 1.23 × 10^4^ and *B* = 1.02 are the coefficients with a correlation coefficient *R* of 0.991; [MeOH vapor] is the concentration of MeOH vapor in ppm. This calibration curve allowed us to determine the dynamic range of the MeOH bio-sniffer as 0.32–20 ppm. Here, the limit of quantification (LOQ) was calculated from ten times the standard deviation of the sensor baseline signal and Equation (1). The obtained dynamic range was found to encompass MeOH concentration in exhaled breath (0.10–2.3 ppm) [46], and therefore, the measurement of MeOH in exhaled breath by the MeOH bio-sniffer was highly possible.

A characteristics comparison of the MeOH bio-sniffer with various reported sensors is shown in Table 1. It shows the high sensitivity of the bio-sniffer among the state-of-the-art MeOH gas sensors.

### 3.3. Measurement of MeOH in Exhaled Breath

**Figure 6:**
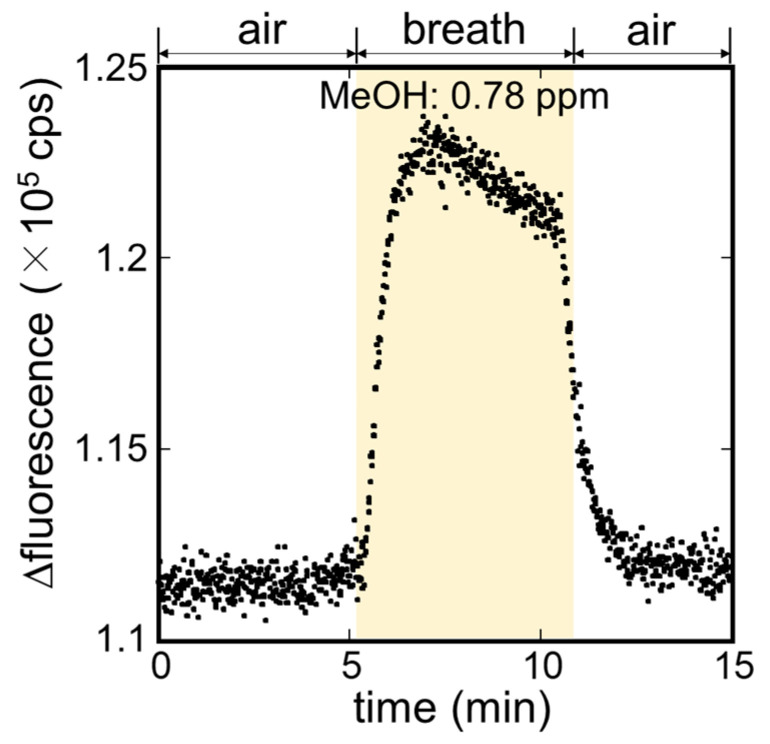
Sensor response to exhaled breath from a healthy subject.

The measurement of MeOH in exhaled breath of a healthy subject was carried out using the MeOH bio-sniffer. The sensorgram during the measurement is presented in Figure 6. The observed sensorgram to the exhaled breath was similar to that of the standard MeOH vapor (see Figure 5a). The fluorescence intensity increased upon pumping the breath sample to the bio-sniffer; then it returned to the baseline when changing breath sample to the filtered air. The concentration of MeOH in the exhaled breath sample was calculated from the average fluorescence intensity of the last 30 s of the breath sample application and equation 1, and it was of 0.78 ppm. The consistency of this value with the concentration range reported by other studies (0.10–2.3 ppm) [46] supported the validity of the measurement.

According to our previous study regarding the AOD-FALDH fluorosensor, the cascade reaction with AOD and FALDH allowed for selective discrimination of methanol from other aliphatic alcohols including ethanol, 1-propanol, 1-butanol, and 2-propanol [36]. On the other hand, it was also observed that use of FALDH lead to reaction with aldehydes such as acetaldehyde and formaldehyde. However, according to the reports by Čáp et al., concentrations of those aldehydes in healthy subject’s breath (acetaldehyde: 7 ppb; formaldehyde: 1 ppb) are much lower than that of MeOH (189 ppb) [46]. Therefore, we have concluded that the signal observed in this experiment was derived from MeOH.

## 4. Conclusions

In this study, we have developed a biochemical gas sensor (bio-sniffer) for MeOH using a cascade reaction with AOD and FALDH for the non-invasive assessment of intestinal flora. After a series of optimizations including FALDH, pH of buffer solution, and methods to prevent water leak from the AOD-FALDH membrane, the dynamic range of the MeOH bio-sniffer was found to be 0.32–20 ppm. This high sensitivity along with the known superior selectivity to MeOH with the cascade reaction suggested the possibility of breath MeOH measurement. In the measurement of exhaled breath sample from a healthy subject, a sensorgram similar to that with standard MeOH vapor was observed. The calculated MeOH concentration in the exhaled breath was 0.78 ppm. The consistency of this value to the reported concentration supported the validity of the measurement and the capability of the MeOH bio-sniffer. The characterizations and a preliminary demonstration with exhaled MeOH in this study indicate the utility and feasibility of the bio-sniffer in the breath MeOH measurement. In future study, we will perform deeper statistical analysis by increasing the number of subjects and investigate the correlation of exhaled methanol and intestinal flora status using the MeOH bio-sniffer to explore the possibility of non-invasive assessment of intestinal flora by exhaled MeOH.

## Figures and Tables

**Table 1:** Comparison of the proposed MeOH bio-sniffer with various reported sensors.

Technology	Material	Dynamic Range	Operating Temp.	Ref.
Chemoresistive	Pd-SnO_2_ nanoparticles	1–1000 ppm	350 °C	[33]
Substrate integrated waveguide	Polyindole	5–500 ppm	RT	[47]
Split ring resonator	Carbon nanotube coated carbon fiber	10–300 ppm	RT	[48]
Chemoresistive	Ce-doped In_2_O_3_ porous nanospheres	2–1000 ppm	320 °C	[49]
Amperometric	Platinum decorated mesoporous TiN	10–300 ppm	RT	[50]
Biofluorescence	AOD-FALDH	0.32–20 ppm	RT	This work

## References

[1] Larsen N., Vogensen F.K., Van Den Berg F.W.J., Nielsen D.S., Andreasen A.S., Pedersen B.K., Al-Soud W.A., Sørensen S.J., Hansen L.H., Jakobsen M. (2010). Gut microbiota in human adults with type 2 diabetes differs from non-diabetic adults. PLoS ONE.

[2] Ravcheev D.A., Godzik A., Osterman A.L., Rodionov D.A. (2013). Polysaccharides utilization in human gut bacterium Bacteroides thetaiotaomicron: Comparative genomics reconstruction of metabolic and regulatory networks. BMC Genom..

[3] Ley R.E., Peterson D.A., Gordon J.I. (2006). Ecological and Evolutionary Forces Shaping Microbial Diversity in the Human Intestine. Cell.

[4] Savage D.C. (1977). Microbial Ecology of the Gastrointestinal Tract. Annu. Rev. Microbiol..

[5] Whitman W.B., Coleman D.C., Wiebe W.J. (1998). Prokaryotes: The unseen majority. Proc. Natl. Acad. Sci. USA.

[6] Sekirov I., Russell S.L., Antunes L.C.M., Finlay B.B. (2016). Gut Microbiota in Health and Disease Gut Microbiota in Health and Disease. Physiol. Rev..

[7] Hooper L.V., Stappenbeck T.S., Hong C.V., Gordon J.I. (2003). Angiogenins: A new class of microbicidal proteins involved in innate immunity. Nat. Immunol..

[8] Achilefu A., Joshi K., Meier M., Mccarthy L.H., Medicine F., Program R., City O. (2017). The influence of gut microbiota on drug metabolism and toxicity. Expert Opin. Drug Metab. Toxicol..

[9] Turnbaugh P.J., Ley R.E., Mahowald M.A., Magrini V., Mardis E.R., Gordon J.I. (2006). An obesity-associated gut microbiome with increased capacity for energy harvest. Nature.

[10] Drasar B.S. (1967). Cultivation of anaerobic intestinal bacteria. Pathology.

[11] Mata L.J., Carrillo C., Villatoro E. (1969). Fecal Microflora in Healthy Persons in a Preindustrial Region. Appl. Microbiol..

[12] Ignyś I., Szachta P., Gałęcka M., Schmidt M., Pazgrat-Patan M., Pazgrat-Patan M. (2014). Methods of analysis of gut microorganism—Actual state of knowledge. Ann. Agric. Environ. Med..

[13] Bäckhed F., Ley R.E., Sonnenburg J.L., Peterson D.A., Gordon J.I. (2005). Host-bacterial mutualism in the human intestine. Science.

[14] Abubucker S., Segata N., Goll J., Schubert A.M., Izard J., Cantarel B.L., Rodriguez-Mueller B., Zucker J., Thiagarajan M., Henrissat B. (2012). Metabolic reconstruction for metagenomic data and its application to the human microbiome. PLoS Comput. Biol..

[15] Dorokhov Y.L., Shindyapina A.V., Sheshukova E.V., Komarova T.V. (2015). Metabolic Methanol: Molecular Pathways and Physiological Roles. Physiol. Rev..

[16] Martens E.C., Lowe E.C., Chiang H., Pudlo N.A., Wu M., McNulty N.P., Abbott D.W., Henrissat B., Gilbert H.J., Bolam D.N. (2011). Recognition and degradation of plant cell wall polysaccharides by two human gut symbionts. PLoS Biol..

[17] Siragusa R.J., Cerda J.J., Baig M.M., Burgin C.W., Robbins F.L. (1988). Methanol production from the degradation of pectin by human colonic bacteria. Am. J. Clin. Nutr..

[18] Laakso O., Haapala M., Jaakkola P., Laaksonen R., Luomanmäki K., Nieminen J., Pettersson M., Päivä H., Räsänen M., Himberg J.J. (2001). FT-IR breath test in the diagnosis and control of treatment of methanol intoxications. J. Anal. Toxicol..

[19] Dorokhov Y.L., Komarova T.V., Petrunia I.V., Kosorukov V.S., Zinovkin R.A., Shindyapina A.V., Frolova O.Y., Gleba Y.Y. (2012). Methanol may function as a Cross-Kingdom signal. PLoS ONE.

[20] Lindinger W., Taucher J., Jordan A., Hansel A., Vogel W. (1997). Endogenous production of methanol after the consumption of fruit. Alcohol. Clin. Exp. Res..

[21] Eriksen S.P., Kulkarni A.B. (1963). Methanol in normal human breath. Science.

[22] Turner C., Španěl P., Smith D. (2006). A longitudinal study of methanol in the exhaled breath of 30 healthy volunteers using selected ion flow tube mass spectrometry, SIFT-MS. Physiol. Meas..

[23] Španěl P., Dryahina K., Vicherková P., Smith D. (2015). Increase of methanol in exhaled breath quantified by SIFT-MS following aspartame ingestion. J. Breath Res..

[24] He C., Liu L., Korposh S., Correia R., Morgan S.P. (2021). Volatile Organic Compound Vapour Measurements Using a Localised Surface Plasmon Resonance Optical Fibre Sensor Decorated with a Metal-Organic Framework. Sensors.

[25] Kittle J., Fisher B., Kunselman C., Morey A., Abel A. (2019). Vapor Selectivity of a Natural Photonic Crystal to Binary and Tertiary Mixtures Containing Chemical Warfare Agent Simulants. Sensors.

[26] Furuuchi N., Shrestha R., Yamashita Y., Hirao T., Ariga K., Shrestha L. (2019). Self-Assembled Fullerene Crystals as Excellent Aromatic Vapor Sensors. Sensors.

[27] Slobodian P., Riha P., Olejnik R., Matyas J., Slobodian R. (2021). Microstrip Resonant Sensor for Differentiation of Components in Vapor Mixtures. Sensors.

[28] Blank A., Guendelman G., Linzon Y. (2020). Vapor Sensing with Polymer Coated Straight Optical Fiber Microtapers Based on Index Sensitive Interference Spectroscopy of Surface Stress Birefringence. Sensors.

[29] Angulo Barrios C. (2019). Scotch Tape Optical Vapor Sensor for Ethanol–Methanol Mixtures. Sensors.

[30] Li Y., Deng D., Chen N., Xing X., Xiao X., Wang Y. (2016). Enhanced methanol sensing properties of SnO_2_ microspheres in a composite with Pt nanoparticles. RSC Adv..

[31] Chen Y., Dong Z., Xue X., Chen S., Natan A., Lv Y., Chen C., Yang Y.Y., Cen W., Yang Y.Y. (2020). High-sensitivity and high-selectivity detection of methanol based on La-doped SnO_2_ sensor. Appl. Phys. A Mater. Sci. Process..

[32] Andrés M.A., Vijjapu M.T., Surya S.G., Shekhah O., Salama K.N., Serre C., Eddaoudi M., Roubeau O., Gascón I. (2020). Methanol and Humidity Capacitive Sensors Based on Thin Films of MOF Nanoparticles. ACS Appl. Mater. Interfaces.

[33] van den Broek J., Abegg S., Pratsinis S.E., Güntner A.T. (2019). Highly selective detection of methanol over ethanol by a handheld gas sensor. Nat. Commun..

[34] Güntner A.T., Magro L., van den Broek J., Pratsinis S.E. (2021). Detecting methanol in hand sanitizers. iScience.

[35] Hayasaka T., Lin A., Copa V.C., Lopez L.P., Loberternos R.A., Ballesteros L.I.M., Kubota Y., Liu Y., Salvador A.A., Lin L. (2020). An electronic nose using a single graphene FET and machine learning for water, methanol, and ethanol. Microsyst. Nanoeng..

[36] Toma K., Iwasaki K., Arakawa T., Iwasaki Y., Mitsubayashi K. (2021). Sensitive and selective methanol biosensor using two-enzyme cascade reaction and fluorometry for non-invasive assessment of intestinal bacteria activity. Biosens. Bioelectron..

[37] Mansour E., Vishinkin R., Rihet S., Saliba W., Fish F., Sarfati P., Haick H. (2020). Measurement of temperature and relative humidity in exhaled breath. Sens. Actuators B Chem..

[38] Chien P.-J., Suzuki T., Tsujii M., Ye M., Toma K., Arakawa T., Iwasaki Y., Mitsubayashi K. (2017). Bio-sniffer (gas-phase biosensor) with secondary alcohol dehydrogenase (S-ADH) for determination of isopropanol in exhaled air as a potential volatile biomarker. Biosens. Bioelectron..

[39] Iitani K., Chien P.-J., Suzuki T., Toma K., Arakawa T., Iwasaki Y., Mitsubayashi K. (2018). Fiber-Optic Bio-sniffer (Biochemical Gas Sensor) Using Reverse Reaction of Alcohol Dehydrogenase for Exhaled Acetaldehyde. ACS Sens..

[40] Chien P.-J.J., Suzuki T., Tsujii M., Ye M., Minami I., Toda K., Otsuka H., Toma K., Arakawa T., Araki K. (2017). Biochemical Gas Sensors (Biosniffers) Using Forward and Reverse Reactions of Secondary Alcohol Dehydrogenase for Breath Isopropanol and Acetone as Potential Volatile Biomarkers of Diabetes Mellitus. Anal. Chem..

[41] Toma K., Suzuki S., Arakawa T., Iwasaki Y., Mitsubayashi K. (2021). External ears for non-invasive and stable monitoring of volatile organic compounds in human blood. Sci. Rep..

[42] Kudo H., Yagi T., Chu M.X., Saito H., Morimoto N., Iwasaki Y., Akiyoshi K., Mitsubayashi K. (2008). Glucose sensor using a phospholipid polymer-based enzyme immobilization method. Anal. Bioanal. Chem..

[43] Toma K., Tsujii M., Arakawa T., Iwasaki Y., Mitsubayashi K. (2021). Dual-target gas-phase biosensor (bio-sniffer) for assessment of lipid metabolism from breath acetone and isopropanol. Sens. Actuators B Chem..

[44] Arakawa T., Suzuki T., Tsujii M., Iitani K., Chien P.-J.J., Ye M., Toma K., Iwasaki Y., Mitsubayashi K. (2019). Real-time monitoring of skin ethanol gas by a high-sensitivity gas phase biosensor (bio-sniffer) for the non-invasive evaluation of volatile blood compounds. Biosens. Bioelectron..

[45] Hesse C., Schulz F., Bull C.T., Shaffer B.T., Yan Q., Shapiro N., Hassan K.A., Varghese N., Elbourne L.D.H., Paulsen I.T. (2018). Genome-based evolutionary history of *Pseudomonas* spp.. Environ. Microbiol..

[46] Čáp P., Dryahina K., Pehal F., Španěl P. (2008). Selected ion flow tube mass spectrometry of exhaled breath condensate headspace. Rapid Commun. Mass Spectrom..

[47] Kumar A., Wang C., Meng F.Y., Jiang C.P., Yan G.F., Zhao M., Jing C.Q., Wang L. (2020). Ultrafast Detection and Discrimination of Methanol Gas Using a Polyindole-Embedded Substrate Integrated Waveguide Microwave Sensor. ACS Sens..

[48] Singh S.K., Azad P., Akhtar M.J., Kar K.K. (2018). Improved Methanol Detection Using Carbon Nanotube-Coated Carbon Fibers Integrated with a Split-Ring Resonator-Based Microwave Sensor. ACS Appl. Nano Mater..

[49] Han D., Song P., Zhang S., Zhang H., Xu Q., Wang Q. (2015). Enhanced methanol gas-sensing performance of Ce-doped In2O3 porous nanospheres prepared by hydrothermal method. Sens. Actuators B Chem..

[50] Meng D., Zhang S., Thomas T., Zhao R., Shi Y., Qu F., Yang M. (2020). Platinum decorated mesoporous titanium nitride for fuel-cell type methanol gas sensor. Sens. Actuators B Chem..

